# Supplementary material for: CESCR: CP-ABE for efficient and secure sharing of data in collaborative ehealth with revocation and no dummy attribute
Source: PLoS One. 2021 May 11;16(5):e0250992. doi: 10.1371/journal.pone.0250992 (PMC8112809; doi:10.1371/journal.pone.0250992)
Supplement: Attachment — Submitted filename: Response to Reviewers.doc [file pone.0250992.s001.doc]

**Manuscript ID:** PONE-D-21-04536

**Manuscript Title:** “CESCR: CP-ABE for Efficient and Secure Sharing of Data in Collaborative eHealth with Revocation and no Dummy Attribute”

**To:** **PLOS ONE** Editor

**Re:** Response to reviewer

Dear Editor,

Thank you for allowing a re-submission of our manuscript, with an opportunity to address the reviewer’s comments.

We are uploading (a) our point-by-point response to the comments (below) (response to reviewers), (b) an updated manuscript with yellow highlighting indicating changes, and (c) a clean updated manuscript without highlights (PDF main document).

Best regards,

Jong Wook Kim (Corresponding Author)

**Reviewer#1, Concern # 1:** *The paper lacks innovative points. The fifth point of user anti-collusion is actually something that has already been mentioned in the first point, and the attribute/user cancellation mentioned by the author is only the technology of other solutions. The fourth point of innovation is also a feature of the OBDD access structure itself, rather than unique to the solution.*

**Author response:** We thank the reviewer for the concern. We believe we have made a number of innovative points in this work. (1) To the best of our knowledge, we believe the idea of outsourcing encryptions and decryptions without a dummy attribute is a novel contribution. And, (2) Limiting the attribute elements in the ciphertext to those only associated with attribute groups is another innovation that we have not come across in the previous studies. Thus, by combining these innovative points with existing ideas, we are able to design a CP-ABE scheme that can achieve collusion resistance, unboundedness, revocation, improved efficiency and expressiveness during health data sharing in collaborative ehealth systems.

**Reviewer#1, Concern # 2:** *In the section of Security Analysis, the probability of challenger B's success has not been analyzed in detail. Please use the mathematical formula to analyze concretely.*

**Author response:** We thank the reviewer for pointing out this weakness. In the revised manuscript, we expounded on the probabilistic success of the challenger B. The detailed analysis is presented in section 6 of the revised manuscript.

**Reviewer#1, Concern # 3:** *In the section of Simulation and Performance Analysis, the authors only chose to compare with one plan, which makes the degree of persuasion was not strong.*

**Author response:** We apologize for not including more schemes for comparison in the original manuscript. In the revised manuscript, we simulated one additional scheme for comparison with our scheme. The results are included in section 7.2 of the revised manuscript.

**Reviewer#1, Concern # 4:** *The content format and reference format of this article are not accurate enough. Please double check and correct.*

**Author response:** We apologize for the formatting mistakes. During the manuscript revision, we used the PLOS ONE template and sample published articles to help to reformat our manuscript. We also used the “plos2015.bst” file to generate the references for inclusion in the revised manuscript.

**Reviewer#2, Concern # 1:** *The introduction part is well written.*

**Author response:** We thank the reviewer for appreciating our effort.

**Reviewer#2, Concern # 2:** *The technical novelty of the paper is good. The research problem and research methods are described clearly. The paper is potentially worthy of publication.*

**Author response:** We thank the reviewer for the positive comment.

**Reviewer#2, Concern # 3:**  *The related work section is comprehensive. However, the authors are requested to analyse the following papers in the releted work section.
* An efficient anonymous authentication and confidentiality preservation schemes for secure communications in wireless body area networks, wireless networks.
* Efficient and secure anonymous authentication with location privacy for IoT-based WBANs, IEEE Transactions on Industrial Informatics.*

**Author response:** We thank the reviewer for the suggestion. In the “related works” section of the revised manuscript, we cited the suggested articles as [31] and [32].

**Reviewer#2, Concern # 4:** *The contribution of this paper is well.*

**Author response:** We thank the reviewer for acknowledging our contribution.
